# Mean platelet volume: a new predictor of ischaemic stroke risk in patients with nonvalvular atrial fibrillation

**DOI:** 10.1186/s12872-020-01525-x

**Published:** 2020-05-20

**Authors:** Meifang Zheng, Shean Chen, Ye Zhu, Xiang Gu

**Affiliations:** 1 Clinical Medical College, Yangzhou University, Yangzhou, Jiangsu China; 2 Department of Cardiology, Northern Jiangsu People’s Hospital, No. 98, Nantong West Road, Yangzhou, 225001 Jiangsu China

**Keywords:** Atrial fibrillation, Mean platelet volume, Ischaemic stroke, CHA2DS2-VASc score

## Abstract

**Background:**

Mean platelet volume (MPV) has been identified as an individual risk factor for stroke and thrombosis. Concurrently, ischaemic stroke caused by nonvalvular atrial fibrillation (AF) has attracted increasing attention. The aim of this study was to investigate the association between MPV and the risk of ischaemic stroke in AF patients not receiving anticoagulant therapy.

**Methods:**

A total of 370 patients with nonvalvular AF were enrolled. Patients were divided into a control group and a stroke group according to the presence of ischaemic stroke.

**Results:**

The MPV level and CHA2DS2-VASc scores of the stroke group were higher than those of the control group (all *p* < 0.001). The ischaemic stroke event rates were significantly increased in the highest MPV tertile when compared to the lowest MPV tertile (56.9% vs. 30.3%, *p* < 0.001). Multivariate logistic regression analysis showed that CHA2DS2-VASc, MPV and D-dimer (D2) were predictors of ischaemic stroke [all *p* < 0.05]. The receiver operating characteristic (ROC) curve analysis indicated that an MPV value of 11.65 fL could predict ischaemic stroke with a sensitivity of 67.3% and specificity of 58.5%, while a CHA2DS2-VASc score cutoff value 3.5 had a sensitivity of 52.1% and specificity of 87.3%. The predictive value of the combined model of CHA2DS2-VASc+MPV was higher than others (comparison calculated by using MedCalc software). The sensitivity of the CHA2DS2-VASc score combined with MPV for predicting ischaemic stroke was 72.1%, and the specificity was 81.5%.

**Conclusions:**

MPV could be a new predictor of ischaemic stroke risk in patients with AF. Moreover, the CHA2D2S2-VASc combined with MPV can improve predictive value with higher sensitivity and it could be a powerful tool for stratifying patients in terms of ischaemic stroke risk.

## Background

Accurate ischaemic stroke risk stratification in atrial fibrillation (AF) patients is an important aspect of AF management. The CHA2DS2-VASc (congestive heart failure, hypertension, age ≥ 75 years, diabetes mellitus, stroke or transient ischaemic stroke (TIA) or other embolic events, vascular disease, age 65 to 74 years, sex category) score is the most widely used AF-related ischaemic stroke risk stratification tool in the world today. It was recommended as the only risk stratification tool for ischaemic stroke in patients with AF by the 2016 ESC Guidelines [1] and the 2019 AHA/ACC/HRS Guidelines [2].

Studies have shown that thromboembolic events in patients with AF are associated with the presence of a prothrombotic state (PTS) [3]. PTS refers to a pathological condition caused by various factors, such as haemostasis, coagulation and anticoagulant system abnormalities. Platelet activation is a component of PTS, and markers of platelet activation include mean platelet volume (MPV), soluble P-selectin, platelet factor 4, and glycoprotein IIb/IIIa [4]. MPV is an accurate marker of platelet size and is a routine item in the analysis of whole blood counts. It is more convenient and economical to measure than other platelet activation markers. Studies have shown that a high MPV level was independently associated with thrombosis [5, 6]. Furthermore, some studies have shown that MPV can be used to predict ischaemic stroke risk in patients with AF [7–9]. It was especially suitable for patients with AF with a low to intermediate traditional thromboembolic risk (CHADS2 score < 2, congestive heart failure, hypertension, age ≥ 75 years, diabetes mellitus, stroke), which has a complementary role in the prevention of ischaemic stroke in AF patients [10].

In the previous study, the researchers did not stratify AF patients with or without anticoagulant treatment. However, due to the differences in drug-related genes and the lack of regular monitoring of the international normalized ratio in the East Asian region, especially in China, the anticoagulation intensity among patients in these studies may differ. In this study, we focused on patients with AF who were not treated with anticoagulant therapy, which can reduce the error caused by different strengths of anticoagulation. We explored the risk factors of AF-related ischaemic stroke in the Chinese population and analysed the value of MPV, as well as the CHA2DS2-VASc score combined with MPV, in predicting AF-related ischaemic stroke risk to provide effective guidance for the prevention and management of AF-related ischaemic stroke.

## Methods

### Study population

A total of 370 patients with nonvalvular AF who underwent MPV measurement at our hospital between January 2017 and December 2018 were included in this study. Verbal informed consent were obtained from all involved participants, and the study was conducted according to the Helsinki Declaration. The study was approved by the ethics committee of Northern Jiangsu People’s Hospital. We included patients between the ages of 18 and 80 years who were not using any anticoagulation drugs. Patients with valvular heart disease, congenital heart disease, thyroid disease, malignant tumours, autoimmune disease, severe infection, blood system disease, connective tissue disease, rheumatic activity, pulmonary embolism, deep vein thrombosis, severe liver and kidney dysfunction, electrolyte imbalance, history of traumatic surgery or acute myocardial infarction within the past 3 months combined with other types of arrhythmias, were excluded. The included patients were followed up until December 31, 2019, they were divided into two groups according to the presence of acute ischaemic stroke during the follow-up period. The diagnosis of acute ischaemic stroke conformed to the 2014 “Guidelines for the Diagnosis and Treatment of Acute Ischaemic Stroke in China” [11]. The diagnostic criteria were as follows: (1) acute onset; (2) regional neurologic impairment; (3) the duration of symptoms or signs is not limited (when imaging shows that there is a responsible ischaemic lesion) or lasts for more than 24 h (when imaging shows that there is no responsible lesion); (4) nonvascular causes have been excluded; and (5) cerebral CT/MRI excluded cerebral haemorrhage. Besides, all patients included in the stroke group were excluded from the possibility of stroke from other causes, like patent foramen ovale tested by echocardiography, prominent carotid artery stenosis tested by carotid duplex ultrasound, and cerebral arterial stenosis tested by TCD (intracranial dopley). The possibility of cardioembolism was considered by doctors to be the greatest with the evidence of AF in ECG or Holter. The control group consisted of 205 patients with AF, and the stroke group consisted of 165 patients with AF-related ischaemic stroke. All patients in the stroke group were recorded with atrial fibrillation by electrocardiogram prior to stroke.

### Data collection

The clinical data of all patients were collected at the time of the first diagnosis of AF, including basic demographic characteristics, previous disease history, basic medication use, MPV and other items included within the whole blood count, fibrinogen (FIB), D-dimer (D2), creatinine (Cr) and uric acid (UA), left atrial diameter (LAD) and left ventricular ejection fraction (LVEF). All the blood test results were from the first venous blood test results after admission and prior to any treatment. The CHA2DS2-VASc scores of all patients were calculated. The neutrophil-lymphocyte ratio (NLR) is the neutrophil count divided by the lymphocyte count. Vascular disease refers to myocardial infarction, complex aortic plaque, and peripheral artery disease including revascularization, amputation because of peripheral artery disease, or angiographic evidence of peripheral artery disease.

### Statistical analysis

Data were analysed with SPSS software version 20.0 for Windows (SPSS Inc., Chicago, Illinois, USA). Continuous variables with a normal distribution were described by the mean ± standard deviation, and continuous variables without a normal distribution were described by quartile. The categorical variables were summarized as frequencies and percentages. Categorical variables were compared by the chi-squared test or Fisher’s exact test. Comparisons between two continuous variables with normal distribution were carried out with the independent samples Student’s t-test, and comparisons between two continuous variables without normal distributions were carried out with the Mann-Whitney U test. Univariate and multivariate binary logistic regression analysis with the backward likelihood ratio method were employed to determine the predictors of ischaemic stroke. Receiver operating characteristic (ROC) curve analysis was performed to determine the sensitivity and specificity with a 95% confidence interval (CI) for the MPV and CHA2DS2-VASc at cutoff values calculated by the Youden index. Medcalc software was used to compare different ROC curves. A two-tailed *p* value of less than 0.05 was considered statistically significant.

## Results

### Clinical characteristics

A total of 370 patients with nonvalvular AF were enrolled, including 206 males (55.7%), and the mean age of all patients was 68.16 ± 8.75 years. A total of 205 patients were included in the control group, and 165 patients were included in the stroke group. The clinical characteristics of the two groups are shown in Table 1. The two groups were significantly different in terms of age as well as in history of heart failure, hypertension, diabetes, ischaemic stroke or transient ischaemic stroke and vascular disease (*p*<0.05 for all variables). The blood test and echocardiography results of the two groups are shown in Table 1. The MPV level of the stroke group was higher than that of the control group (12.08 ± 1.05 vs. 11.42 ± 1.08 fL, *p* < 0.001). The CHA2DS2-VASc score of the stroke group was 2.07 ± 1.31, while the score of the control group was 2.04 ± 1.31, and the difference was statistically significant (*p* < 0.001).

**Table 1:** Baseline characteristics of the two groups

Variables	Control group(*n* = 205)	Stroke group(*n* = 165)	t/Z /χ2 value	*p* value
Male (%)	117 (57.1)	89 (53.9)	0.364	0.546
Age (years old)	65.40 ± 9.48	71.58 ± 6.24	5.087	<0.001***
BMI (kg/m2)	24.34 ± 3.12	24.48 ± 3.64	0.802	0.690
Smoke (%)	41 (20.0)	34 (20.6)	0.021	0.885
Drink (%)	27 (13.2)	29 (17.6)	1.381	0.240
Heart failure (%)	5 (2.4)	14 (8.5)	6.859	0.009**
Hypertension (%)	105 (51.2)	120 (72.7)	17.745	<0.001***
Diabetes (%))	22 (10.7)	38 (23.0)	10.177	0.001**
Stroke/TIA (%)	13 (6.3)	49 (29.7)	35.750	<0.001***
Vascular disease (%)	16 (7.8)	24 (14.5)	4.308	0.038**
COPD (%)	3 (1.5)	3 (1.8)	0.072	0.789
Gout (%)	4 (2.0)	1 (0.6)	1.352	0.386
CCB (%)	40 (19.5)	32 (19.4)	0.001	0.977
Statins (%)	11 (5.4)	12 (7.3)	0.570	0.450
Diuretic (%)	8 (3.9)	2 (1.2)	2.516	0.195
ACEI/ARB (%)	49 (23.9)	26 (15.8)	3.753	0.053
Digoxin (%)	4 (2.0)	4 (2.4)	0.097	0.756
Antiplatelet drugs (%)	23 (11.2)	30 (18.2)	3.611	0.057
β-blocker (%)	34 (16.6)	16 (9.7)	3.712	0.054
RBC (× 10^12^/L)	4.54 ± 0.55	4.59 ± 0.52	0.848	0.397
HGB (g/L)	139.76 ± 15.79	139.27 ± 15.83	0.295	0.768
HCT (%)	41.72 ± 4.55	41.57 ± 4.29	0.325	0.746
MCHC (g/L)	3.35 ± 0.128	3.34 ± 0.135	0.140	0.889
MCV (fl)	91.95 ± 6.18	90.73 ± 5.05	2.030	0.043*
RDW-CV (%)	13.42 ± 2.37	13.67 ± 2.25	1.032	0.303
WBC (×10^9^/L)	6.45 ± 2.01	7.22 ± 2.50	3.302	0.001**
PLT (×10^9^/L)	176.01 ± 51.73	171.38 ± 53.15	0.845	0. 399
MPV (fl)	11.42 ± 1.08	12.08 ± 1.05	5.948	<0.001***
PDW (%)	14.84 ± 3.51	15.91 ± 2.94	3.116	0.002**
FIB (g/L)	2.97 ± 0.91	2.79 ± 0.70	1.916	0.056
D2 (mg/L)	0.36 (0.24–0.81)	0.71 (0.39–1.35)	5.269	<0.001***
UA (μmol/L)	360.91 ± 110.73	349.50 ± 103.19	1.011	0. 313
Cr (μmol/L)	82.37 ± 21.37	73.29 ± 23.09	3.899	<0.001***
LAD (mm)	37.83 ± 6.27	40.2 ± 5.90	3.468	0.001**
LVEF (%)	57.80 ± 7.45	57.04 ± 7.20	0.935	0.350
NLR	2.33 (1.71–3.58)	3.11 (1.94–5.44)	4.017	<0.001***
CHADS2	0.94 ± 0.93	2.07 ± 1.31	27.206	<0.001***
CHA2DS2-VASc	2.04 ± 1.31	3.54 ± 1.56	9.896	<0.001***

* *p*<0.05, ** *p*<0.01, *** *p*<0.001

### Predictors of Ischaemic stroke

Univariate binary logistic regression analysis showed that WBC, MPV, D2, Cr and the CHA2DS2-VASc score were all risk factors (all *p* < 0.01) (Table 2), whereas multivariate binary logistic regression analysis with the backward likelihood ratio method showed that only the CHA2DS2-VASc score (OR = 2.154; 95% CI, 1.739–2.668; *p* < 0.001), MPV (OR = 1.962; 95% CI, 1.493–2.579; *p* < 0.001) and D2 (OR = 1.80; 95% CI, 1.13–2.84; *p* = 0.012) were risk factors for ischaemic stroke in patients with AF (Table 2). (Cr was not further analysed because the Cr OR was 0.98, which means a low correlation between Cr and ischaemic stroke).

**Table 2:** Risk factors for ischemic stroke analyzed by logistic regression

Variables	univariate				multivariate			
	*p* value	OR	95%CI		*p* value	OR	95%CI	
WBC	0.001**	1.167	1.061	1.284	0.081	1.126	0.986	1.286
MPV	<0.001***	1.783	1.450	2.191	<0.001***	1.962	1.493	2.579
D2	0.006**	1.318	1.082	1.606	0.04*	1.230	1.009	1.498
Cr	<0.001***	0.981	0.971	0.991	0.002**	0.980	0.967	0.992
CHA2DS2-VASc	<0.001***	2.029	1.710	2.408	<0.001***	2.154	1.739	2.668

* *p*<0.05, ** *p*<0.01, *** *p*<0.001

### Subgroup analyses by MPV and CHA2DS2-VASc score

**Fig. 1:**
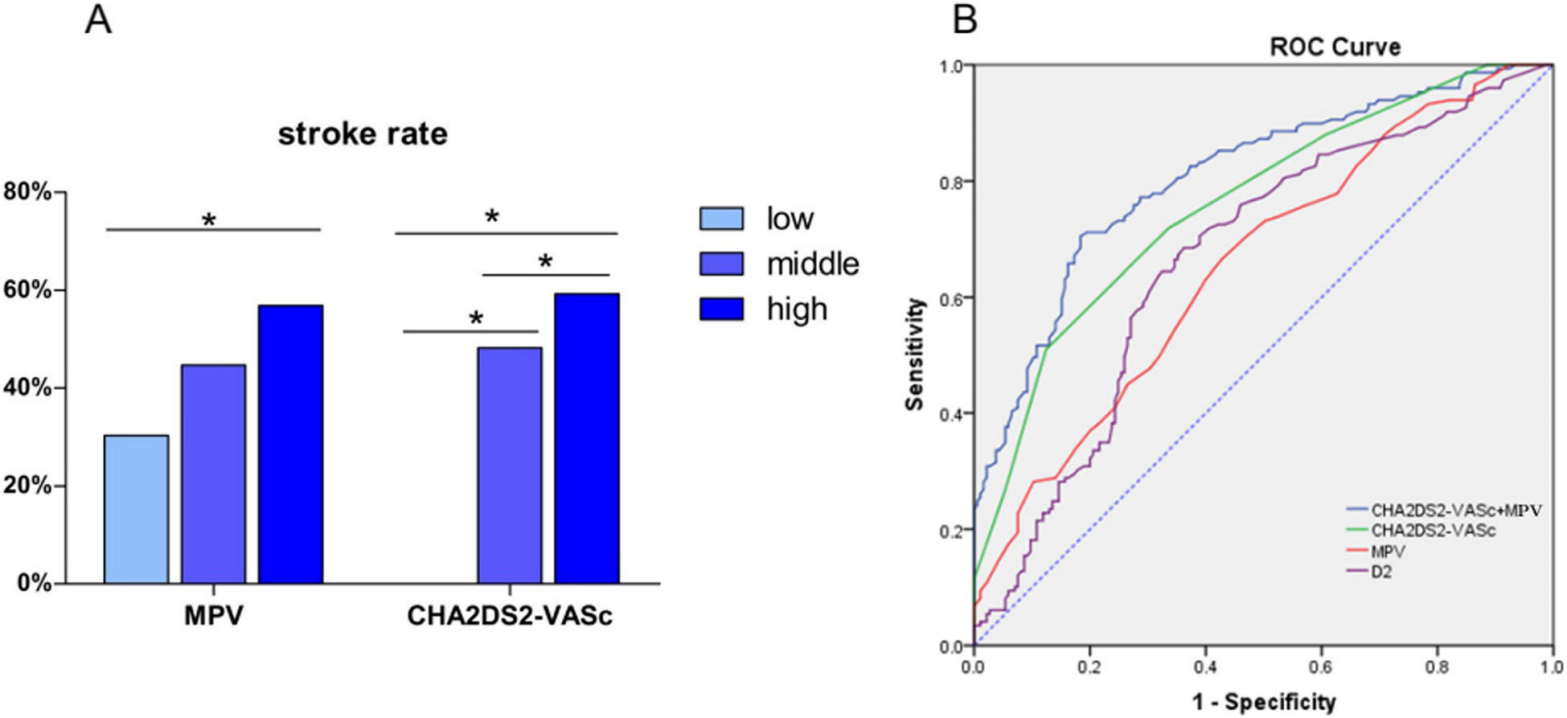
**a** Subgroup Analyses by MPV and CHA2DS2-VASc Score. **b** The Predictive Value of Risk Factors

Patients were stratified into tertiles according to MPV (< 11.2 fL, 11.2–12.2 fL and ≥ 12.2 fL), and a subgroup analysis was performed using the χ2 test. The results showed that the ischaemic stroke event rates increased significantly in the highest MPV tertile group when compared to the lowest MPV tertile group (56.9% vs. 30.3%, *p* < 0.001) (Fig. 1a).

Furthermore, subgroup analyses stratified according to the CHA2DS2-VASc score were performed to further verify the predictive power of the CHA2DS2-VASc score. The results showed that the incidence of ischaemic stroke gradually increased with increasing CHA2DS2-VASc score; in the low-risk group (CHA2DS2-VASc = 0), the middle-risk group (CHA2DS2-VASc = 1) and the high-risk group (CHA2DS2-VASc≥2) the incidence was 0, 26.3, and 53.3%, respectively. The difference was statistically significant. (*p* < 0.05) (Fig. 1a).

### The predictive value of risk factors

The predictive value of risk factors for predicting ischaemic stroke events in patients with AF was evaluated by ROC analysis. The area under the curve (AUC) values of MPV, D-dimer and CHA2DS2-VASc were 0.664, 0.668 and 0.761, respectively (Table 3, Fig. 1b). The results of the ROC analysis indicated a cutoff value of 11.65 fL for MPV with 67.3% sensitivity and 58.5% specificity (*p* < 0.001) for the prediction of ischaemic stroke, while the CHA2DS2-VASc score cutoff value of 3.5 had a sensitivity of 52.1% and specificity of 87.3%. In addition, the cutoff value of D-dimer was 0.485 mg/L; the sensitivity was 68.5%, and the specificity was 63.8%.

**Table 3:** The ROC analysis of risk factors

Risk factors	AUC	*p* value	cut-off	Sensitivity (%)	Specificity (%)
MPV	0.664	<0.001***	11.65	67.3	58.5
D2	0.668	<0.001***	0.485	68.5	63.8
CHA2DS2-VASc	0.761	<0.001***	3.5	52.1	87.3
CHA2DS2-VASc+D2	0.783	<0.001***	0.472	69.1	78.4
CHA2DS2-VASc+MPV	0.812	<0.001***	0.487	72.1	81.5
CHA2DS2-VASc+MPV + D2	0.816	<0.001***	0.479	71.1	81.1

### The predictive ability of combined predictive models

To assess the predictive ability of CHA2DS2-VASc combined with MPV, logistic regression analysis was conducted with group as the dependent variable and CHA2DS2-VASc and MPV as independent variables. The probability value (P) was calculated by SPSS software during regression analysis. The probability value (P) was the test variable, and the group was the state variable in the ROC analysis. The area under the curve (AUC) value for the CHA2DS2-VASc combined with MPV model was 0.812, and the cutoff value, which was calculated by the Youden index, was 0.487, with 72.1% sensitivity and 81.5% specificity (*p* < 0.001) for the prediction of ischaemic stroke (Fig. 1b). Using the same method, we obtained an CHA2DS2-VASc+D2 AUC value of 0.783, a sensitivity of 69.1% and a specificity of 78.4% (*p* < 0.001) (Table 3). Medcalc software was used to compare various ROC curves, and the results showed that the CHA2DS2-VASc+MPV AUC value was higher than that of CHA2DS2-VASc or MPV alone, and the difference was statistically significant (*p* < 0.05) (Table 4). The predictive value of the combined CHA2DS2-VASc+MPV + D2 model was higher than that of CHA2DS2-VASc+D2 (*p* = 0.034) but was similar to that of CHA2DS2-VASc+MPV (*p* = 0.264). In addition, there was no significant difference between the CHA2DS2-VASc+MPV and CHA2DS2-VASc+D2 models (*p* = 0.174); therefore, the combination of the CHA2DS2-VASc score plus MPV had higher sensitivity and specificity and could significantly improve the prediction of ischaemic stroke in patients with AF.

**Table 4:** Comparison of different ROC curves

Different ROC curves	Z value	*p* value
MPV ~ D2	0.296	0.767
CHA2DS2-VASc~ D2	2.595	0.009**
CHA2DS2-VASc~MPV	2.531	0.011*
CHA2DS2-VASc +MPV ~ CHA2DS2-VAS	3.328	<0.001***
CHA2DS2-VASc +MPV ~ MPV	5.565	<0.001***
CHA2DS2-VASc +D2 ~ CHA2DS2-VASc	2.576	0.010*
CHA2DS2-VASc +D2 ~ D2	3.777	<0.001***
CHA2DS2-VASc +MPV ~ CHA2DS2-VASc +D2	1.359	0.174
CHA2DS2-VASc +MPV + D2 ~ CHA2DS2-VASc +MPV	1.118	0.264
CHA2DS2-VASc +MPV + D2 ~ CHA2DS2-VASc +D2	2.123	0.034*

## Discussion

The results suggest that MPV was a risk factor for ischaemic stroke in patients with nonvalvular AF. CHA2DS2-VASc combined with MPV can improve the predictive sensitivity of ischaemic stroke in patients with AF. To the best of our knowledge, this is the first study in China to report that MPV combined with CHA2DS2-VASc scores can improve the predictive value of ischaemic stroke in AF patients.

MPV is a clinical indicator with a normal range of 7–11 fL, which can reflect changes in platelet activation or platelet production. In fact, larger platelets contain denser particles than smaller platelets; they can release more thromboxane A2 and beta-thrombin, and they express more glycoprotein IIb/IIIa receptors. That is, MPV levels are positively correlated with platelet activity and are associated with thrombosis. High MPV levels have been reported to be associated with a variety of cardiovascular and cerebrovascular-related embolic diseases [12–15]. A study published in the European Heart Journal article showed that elevated MPV levels were associated with an increased risk of deep venous thrombosis of the lower extremities and acute myocardial infarction, and increased MPV was considered a predictor of venous thromboembolism [16].

Platelet activity is significantly increased in patients with AF because of the presence of PTS; thus, MPV and other various platelet activity markers are increased [6, 15, 16]. The measurement of other markers (such as soluble P-selectin, platelet factor 4, and glycoprotein IIb/IIIa) is complex, time-consuming, and costly and cannot be routinely applied to normal clinical work. MPV, a routine blood cell analysis index, is easy to obtain in the clinic and in the hospital, and its measurement is inexpensive and can be widely used in clinical work. Several studies have found that increased MPV is a risk factor for ischaemic stroke in patients with AF. Soon-Pyo Hong et al. analysed the risk of ischaemic stroke in 265 patients with AF undergoing ventricular rate or rhythm control and obtained high MPV levels (≥7.85 fL), high CHADS2 scores (≥ 2 points) and lack of ventricular rate control as risk factors for ischaemic stroke in a Cox proportional hazards regression model, independent of sex or anticoagulant therapy [7]. Nermin Bayar et al. divided 90 patients with paroxysmal AF into a symptomatic group and an asymptomatic group based on the presence of ischaemic stroke or TIA. Statistical analysis showed no statistically significant differences between the two groups. The MPV value of the symptomatic group was higher than that of the asymptomatic group (*p* < 0.001). The ROC curve analysis showed that the cutoff value of MPV for predicting ischaemic stroke was 9.85 fL; the sensitivity was 87%, and the specificity was 78% [8]. The results suggested that MPV can be used as a predictor of ischaemic stroke in patients with paroxysmal AF. A study published in 2017 of the relationship between antithrombin III combined with MPV and the risk of ischaemic stroke or left atrial thrombosis in patients with AF indicated that high MPV and lack of antithrombin III were risk factors for ischaemic stroke or left atrial thrombus in patients with AF [17].

The results of this study show that MPV was a risk factor for ischaemic stroke in patients with AF. For every additional unit of MPV, the odds of ischaemic stroke in AF patients increased 1.962 times. In addition, in the subgroup analysis, the higher the MPV value, the higher the proportion of patients with ischaemic stroke (*p* < 0.001). These subgroup analyses further validated the relationship between MPV and the risk of ischaemic stroke at different levels. The ROC curve analysis showed that MPV was a predictor for ischaemic stroke in patients with AF, the AUC was 0.664, and the cutoff value was 11.65 fL, with 67.3% sensitivity and 58.5% specificity for the prediction of ischaemic stroke. This was similar to the results of the above studies abroad, but the cutoff value, AUC value, sensitivity and specificity obtained in this study were slightly different from those of other studies, which may be related to the different blood test methods and reagents, as well as the inclusion and exclusion criteria of the population. In addition, this study was a retrospective study with a relatively small sample size.

The CHA2DS2-VASc score is currently the most widely used AF-related ischaemic stroke risk stratification tool in the world. However, the predictive value of the CHA2DS2-VASc score is at a moderate level [2], and this study found that the AUC was 0.761 with a sensitivity of 52.1% and a specificity of 87.3% when using CHA2DS2-VASc alone to predict ischaemic stroke. However, the AUC was 0.812, with a sensitivity for the CHA2DS2-VASc combined with MPV of 72.1%, which is significantly higher than that of the CHA2D2S2-VASc score alone. It is beneficial to screen patients with AF and a low risk of ischaemic stroke so that timely secondary prevention can be initiated in more patients with increased ischaemic stroke risk to reduce the incidence of ischaemic stroke events.

### Limitations

This study was a single-centre retrospective study. The sample size was limited. Due to regional factors and different detection methods, the results may be different from those conducted in other areas. Multi-centre large-scale studies are needed to reduce this bias. In addition, the patients included in this study were all AF patients who were not receiving anticoagulant therapy. Therefore, we were unable to analyse whether anticoagulant therapy had an effect on biomarkers such as MPV and D-dimer.

## Conclusion

This study concluded that MPV was a risk factor for ischaemic stroke in patients with AF. High MPV (cutoff value of 11.65 fL) can be used to predict ischaemic stroke risk in patients with AF. MPV could be a new predictor of ischaemic stroke risk in patients with AF. Moreover, CHA2DS2-VASc combined with MPV can improve the sensitivity of predicting ischaemic stroke risk, the combination of these two measures could be a powerful risk stratification tool for patients with AF. It will be helpful for identifying patients with low ischaemic stroke risk and increasing secondary prevention of ischaemic stroke in patients with AF.

## Data Availability

The datasets analyzed during the current study are available from the corresponding author on reasonable request.

## Abbreviations

MPV: Mean platelet volume
AF: Atrial fibrillation
ROC: Receiver operating characteristic
TIA: Transient ischemic stroke
PTS: Prothrombotic state
FIB: Fibrinogen
D2: D-dimer
Cr: Creatinine
UA: Uric acid
LAD: Left atrial diameter
LVEF: Left ventricular ejection fraction
NLR: Neutrophil-lymphocyte ratio
CI: Confidence interval
AUC: Area under the curve
